# A case report on dental management of a toddler with Pachygyria

**DOI:** 10.4317/jced.53684

**Published:** 2017-05-01

**Authors:** Vignesh Ravindran, Vishnu Rekha, Sankar Annamalai, Parisa Norouzi-Baghkomeh, Ditto Sharmin

**Affiliations:** 1 B.D.S, Post Graduate student, Department of Pediatric and Preventive Dentistry Meenakshi Ammal Dental College and Hospital, Chennai; 2 M.D.S, Professor and Head of the department, Department of Pediatric and Preventive Dentistry Meenakshi Ammal Dental College and Hospital, Chennai; 3 M.D.S, Reader, Department of Pediatric and Preventive Dentistry Meenakshi Ammal Dental College and Hospital, Chennai; 4 M.D.S, Senior Lecturer, Department of Pediatric and Preventive Dentistry Meenakshi Ammal Dental College and Hospital, Chennai

## Abstract

Children with special health care needs receive less oral care than the normal population, inspite of the high level of dental diseases among them. They are at an increased risk for oral diseases throughout their lifetime. This paper reports a case of a toddler with congenital unusual thick convolutions of the cortex resulting in a condition called pachygyria. Intra oral examination showed multiple abscesses with poor oral hygiene. As the patient was lacking cooperative ability, treatment of full mouth rehabilitation as needed. The parents were advised for regular dental check-ups and informed about maintenance of good oral hygiene. This case report demonstrates the importance of oral hygiene maintenance of special children and also about their short and long term dental treatment protocol for maintaining good oral health.

** Key words:**Pachygyria, general anaesthesia, special child, health care needs, preventive measures.

## Introduction

American Association of Pediatric Dentistry defines special health care needs as “any physical, developmental, mental, sensory, behavioral, cognitive, or emotional impairment or limiting condition that requires medical management, health care intervention, and/or use of specialized services or programs” (1). Such individuals are at an increased risk for oral diseases throughout their lifetime. It has been reported that dental treatment is the greatest unattended health need of the disabled people (2). Pachygyria is a congenital malformation of the cerebral hemisphere resulting in thick convolutions of the cerebral cortex causing seizures, developmental delay, poor muscle control, and feeding/swallowing difficulties (3). The findings presented in this case report are the first to publish the oral features of Pachygyria and emphasizes the early preventive measures in these patients.

## Case Report

A 3-year old male patient reported to the department of Paediatric and Preventive Dentistry with the chief complaint of decayed teeth in lower left and right back teeth region. The patient showed signs of both physical and mental retardation. Medical history revealed that the child was diagnosed with Pachygyria since birth and has seizures, developmental delay, poor muscle tone and control and difficulty with feeding/swallowing. The child was under sodium valproate and clobazam medications and this was the patient’s first dental visit.

**Figure 1:**
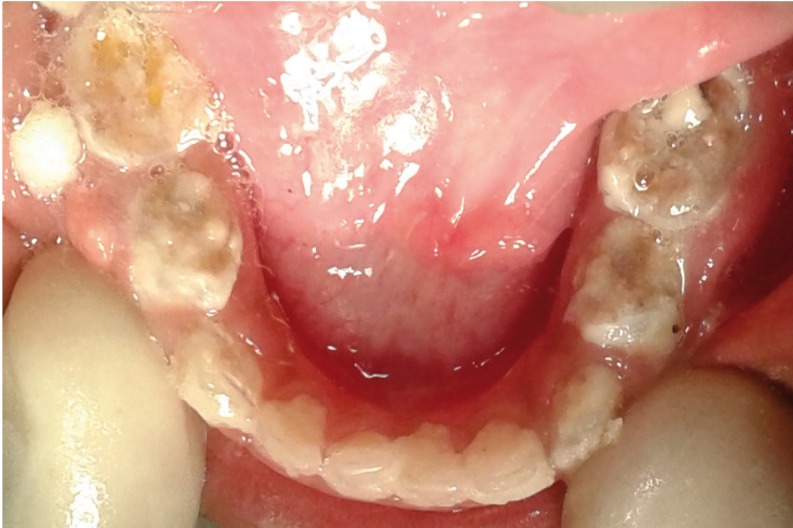
Preoperative clinical image of Mandible.

**Figure 2:**
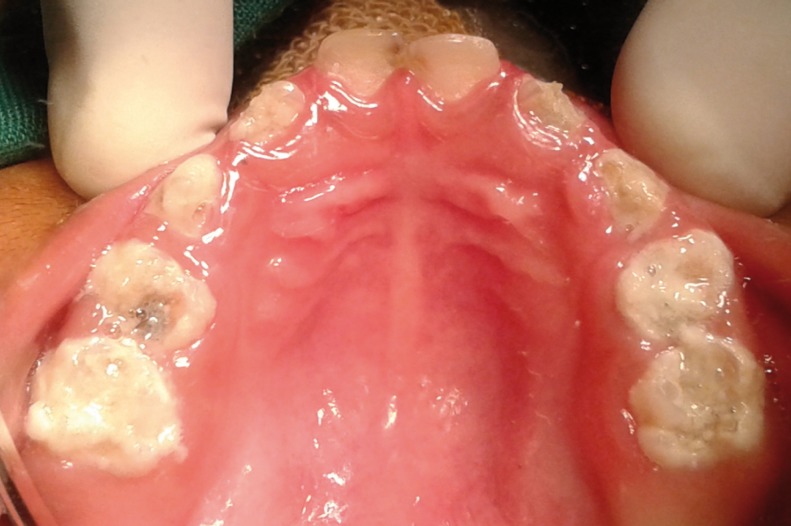
Preoperative clinical image of Maxilla.

**Figure 3:**
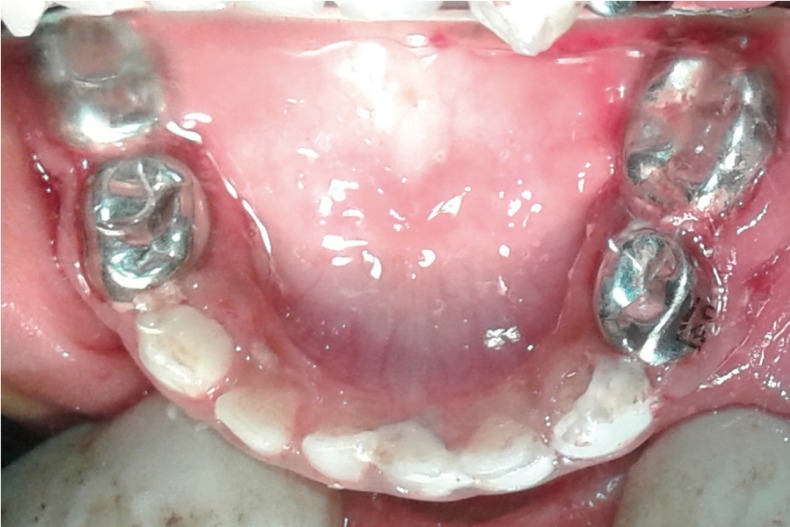
Postoperative clinical image of Mandible.

Since the child fell under lacking cooperative ability according to Wright’s classification (1975), usual clinical and radiographic examinations were not possible on the dental chair. Intra-oral examination was done in knee-to-knee position which revealed extensive carious lesions involving all the primary molars and anterior teeth. Dento-alveolar abscesses were noticed in relation to maxillary right first molar [54], mandibular left and right first molars [74 and 84]. All the teeth were covered with dental plaque and food debris along with minimal gingival enlargement (Figs. 1-3). As dental treatment for such special child was not possible in a regular dental set up, treatment was planned under general anaesthesia with the consent from their paediatrician.

**Figure 4:**
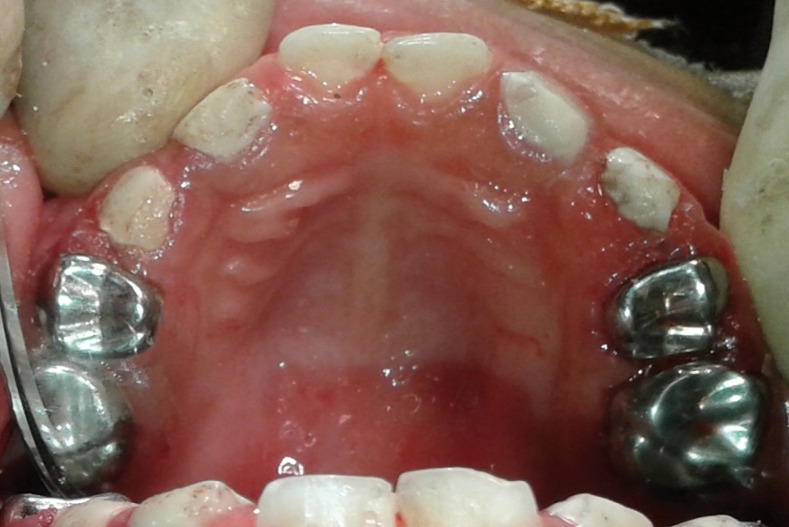
Postoperative clinical image of Maxilla.

Under general anaesthesia oral prophylaxis was done using hand scaling instruments to get a clear view of the carious extensions on all the teeth. Glass ionomer restorations were placed in the maxillary and mandibular anterior teeth [61,73,81,82,83]. Pulpectomy was done followed by composite restorations in maxillary upper right lateral incisor and canine [53 and 52]. Incision and drainage was done in the above mentioned teeth that had dentoalveolar abscesses. Pulpectomy was done followed by stainless steel crowns in the maxillary and mandibular first and second primary molars [54,55,64,65,74,75,84 and 85]. Although the remaining crown structure of the pulpectomised teeth were not optimal, stainless steel crowns were placed to help in mastication (Fig. 4). Recovery from general anesthesia was uneventful and the patient was discharged on the same day. Diet counselling and instructions to maintain proper oral hygiene were given to the parents. Parents were also advised to come for dental check-up every three months; about preventive measures like topical fluoride application every six months; and pit and fissure sealants immediately after the eruption of permanent molars.

## Discussion

The prominent symptoms seen with Pachygyria are the most important etiologic factors causing severe dental caries. Developmental delays make them uncooperative for tooth brushing leading to plaque accumulation and limiting exposure to topical fluorides. They may also be exposed more frequently to medications and sugar sweetened beverages (4). Decreased muscle tone causes stagnation of food along the vestibule providing a long term reduced pH environment. Valproic acid has been linked to gingival overgrowth, apart from phenytoin (5), which increase plaque retentive areas thereby enhancing susceptibility for dental caries and periodontal disease (6). Benzodiazepines have been shown to cause hyposalivation and xerostomia (7) with reduction in stimulated salivary flow rate (8) thereby leading to increased incidence to dental caries.

American Academy of Pediatric Dentistry’s guideline on Caries Risk Assessment have categorised them under High risk for physicians and other non-dental health care providers and Moderate risk for dental providers (9). Attitude and knowledge of the oral health care professionals is of utmost importance while rendering the oral health care to such children (10). The treatment rendered currently provides a short term benefit for the patient. Glass ionomer cement restorations were placed as they release fluoride which may be useful as both preventive and therapeutic approaches (11). Though enough crown structure was not available for proper placement of stainless steel crowns, we intended to do it, as removal of all the primary molars would compromise the mas-tication and also replacement with functional space maintainers is contraindicated in special children. More focus had to be given for long term maintenance of oral health in special children for long term results.

Keeping in mind the importance of prevention, a vigorous approach to preventive measures such as oral hygiene practices, dietary advice and fluoride supplements are required. Dentists should take the responsibility and offer their services in prevention of dental disease in children with special health care needs by establishing communication with the authorities responsible for welfare of these individuals and involve themselves in the total health care (12). Involuntary hand and arm movements or may be partially paralyzed extremities makes caregiver to take responsibility for maintenance of oral hygiene. Caregivers may experience higher levels of stress, which could exacerbate the preceding factors that contribute to poor oral health (13). Therefore, communication should also be established with the parents/care takers of these individuals, and assistance offered in preventive efforts (14). Brushing with a fluoridated dentifrice twice daily should be emphasized to help prevent caries and gingivitis. A non-cariogenic diet should be discussed for long term prevention and if a diet rich in carbohydrates is medically necessary, the dentist should provide strategies to alter frequency besides increasing preventive measures (15).

Management of dental diseases in children with special health care needs should be aimed at both short and long term treatment options. Short term treatment options are focused to control the disease progression and long term options are advised and taught to parents for preventing the recurrence of the disease.
